# Regulation of TFIIIB during F9 cell differentiation

**DOI:** 10.1186/1471-2199-11-21

**Published:** 2010-03-12

**Authors:** Dimitris Athineos, Lynne Marshall, Robert J White

**Affiliations:** 1 Beatson Institute for Cancer Research, Garscube Estate, Switchback Road, Bearsden, Glasgow, G61 1BD, UK

## Abstract

**Background:**

Differentiation of F9 embryonal carcinoma (EC) cells into parietal endoderm (PE) provides a tractable model system for studying molecular events during early and inaccessible stages of murine development. PE formation is accompanied by extensive changes in gene expression both in vivo and in culture. One of the most dramatic is the ~10-fold decrease in transcriptional output by RNA polymerase (pol) III. This has been attributed to changes in activity of TFIIIB, a factor that is necessary and sufficient to recruit pol III to promoters. The goal of this study was to identify molecular changes that can account for the low activity of TFIIIB following F9 cell differentiation.

**Results:**

Three essential subunits of TFIIIB decrease in abundance as F9 cells differentiate; these are Brf1 and Bdp1, which are pol III-specific, and TBP, which is also used by pols I and II. The decreased levels of Brf1 and Bdp1 proteins can be explained by reduced expression of the corresponding mRNAs. However, this is not the case for TBP, which is regulated post-transcriptionally. In proliferating cells, pol III transcription is stimulated by the proto-oncogene product c-Myc and the mitogen-activated protein kinase Erk, both of which bind to TFIIIB. However, c-Myc levels fall during differentiation and Erk becomes inactive through dephosphorylation. The diminished abundance of TFIIIB is therefore likely to be compounded by changes to these positive regulators that are required for its full activity. In addition, PE cells have elevated levels of the retinoblastoma protein RB, which is known to bind and repress TFIIIB.

**Conclusion:**

The low activity of TFIIIB in PE can be attributed to a combination of changes, any one of which could be sufficient to inhibit pol III transcription. Declining levels of essential TFIIIB subunits and of activators that are required for maximal TFIIIB activity are accompanied by an increase in a potent repressor of TFIIIB. These events provide fail-safe guarantees to ensure that pol III output is appropriate to the diminished metabolic requirements of terminally differentiated cells.

## Background

Differentiation of F9 embryonal carcinoma (EC) cells into parietal endoderm (PE) is accompanied by dramatic changes in gene expression. Amongst these are a marked decrease in transcription of tRNA and 5S rRNA genes by pol III, which reflects a reduced requirement for biosynthesis as the differentiating cells stop growing and dividing [1]. Biochemical reconstitution experiments suggested that this change is caused by a specific decrease in activity of the essential pol III transcription factor TFIIIB [1,2]. In support of this, western blot analysis revealed that PE cells express markedly reduced levels of Brf1, a subunit of TFIIIB that binds pol III and brings it to its targets genes [2]. F9 cell differentiation also involves a decrease in the level of the TATA-binding protein TBP, which is another essential subunit of TFIIIB [2,3]. However, the conclusion that pol III transcription is regulated under these circumstances through changes in TFIIIB proved to be controversial. Meissner and colleagues argued that TFIIIB activity is unchanged during differentiation and that transcriptional control reflects other mechanisms [4,5]. They subsequently implcated Bdp1, a third essential subunit of TFIIIB [6]. Here we compare directly the behaviour of the TFIIIB components Brf1, TBP and Bdp1, which had not been done in any of the previous studies. We confirm that each is down-regulated when F9 cells differentiate. Whereas TBP is subject to post-transcriptional control, the fall in Brf1 and Bdp1 levels reflects changes in the corresponding mRNAs. We show that transcriptional repression occurs even if Brf1 expression is maintained in PE cells. We also document changes to several regulators that are known to act directly on TFIIIB.

## Results

### Raising Brf1 expression in F9 cells does not stimulate tRNA expression or proliferation

**Figure 1:**
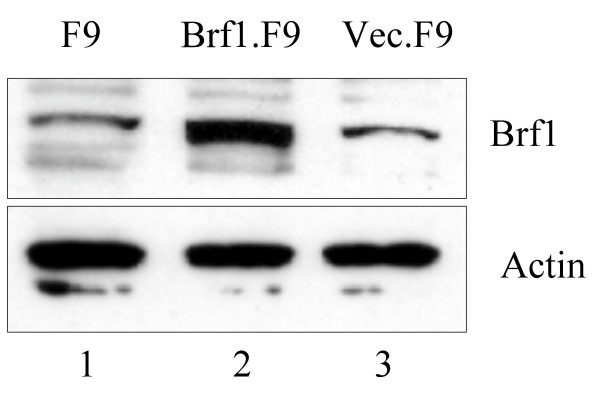
**Expression of exogenous Brf1 in F9 cell stable transfectants**. Western blot of whole cell extract (20 μg) from untransfected F9 cells (lane 1), Brf1.F9 cells (lane 2) and vec.F9 cells (lane 3) probed with antibodies 128 against Brf1 and C-11 against actin, as indicated.

To test the role of Brf1 levels in dictating pol III output during differentiation, we examined whether it is possible to construct an F9 cell derivative in which Brf1 expression is maintained in PE cells. To this end, a pcDNA3 expression vector containing human Brf1 cDNA was introduced by stable transfection to create a cell line that we refer to as Brf1.F9. Since this cDNA is transcribed from a constitutive CMV promoter, it should not be subject to the same regulatory influences that act on the endogenous gene. Indeed, RT-PCR analysis confirmed that expression from the CMV promoter is undiminished following differentiation (data not shown). Quantitation of western blots revealed that the total level of Brf1 in undifferentiated cells was raised by approximately four-fold in Brf1.F9 cells, relative to untransfected cells or control cells, referred to as Vec.F9, that carry empty pcDNA3 vector (Fig. 1).

**Figure 2:**
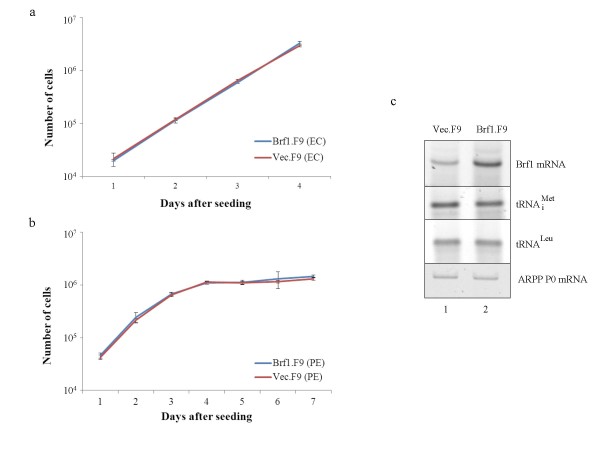
**Stable expression of Brf1 in F9 cells does not stimulate proliferation**. a, b Proliferation curves showing numbers of viable Brf1.F9 (blue) and vec.F9 (red) cells, as determined by trypan blue staining, each day after plating 10^4 ^undifferentiated EC cells (a) or differentiated PE cells (b). Counts were taken in triplicate and the results are presented as averages of three independent experiments +/- standard deviation. c RT-PCR analysis to compare expression of tRNA_i_^Met ^and tRNA^Leu^, and mRNAs encoding Brf1 and ARPP P0 in vec.F9 and Brf1.F9 cells, as indicated.

An increase in Brf1 expression was found to stimulate proliferation of immortalized mouse embryonic fibroblasts (MEFs), IMR90 human diploid fibroblasts and CHO cells [7]. However, the Brf1.F9 cells proliferate at the same rate as matched Vec.F9 control cells, both before and after differentiation (Fig. 2a and 2b). In the case of MEFs, the proliferative response to Brf1 elevation can be mimicked by overexpressing a key Brf1 target, the tRNA_i_^Met ^that is required for translation initiation [7]. Whereas tRNA_i_^Met ^levels increase in response to Brf1 induction in MEFs [7], Brf1.F9 cells express no more tRNA_i_^Met ^than Vec.F9 control cells, after expression was normalised to the mRNA encoding acidic ribosomal phosphoprotein P0 (ARPP P0) (Fig. 2c). As positive control, we confirmed that Brf1 mRNA is elevated in the Brf1.F9 cells. Another tRNA that can be induced in a variety of cell types by raising Brf1 levels is tRNA^Leu ^[7-9]. Nevertheless, as with tRNA_i_^Met^, comparable levels of tRNA^Leu ^are detected in Brf1.F9 and Vec.F9 cells. Although we have only tested two examples, these data suggest that Brf1 is not limiting for tRNA expression in F9 cells. This contrasts with MEFs, HeLa, Rat1a and CHO cells [7-9]. Clearly, a failure to raise expression of key genes offers a ready explanation for the lack of a proliferative response.

### Constant Brf1 expression does not prevent pol III transcription from decreasing when F9 cells differentiate

**Figure 3:**
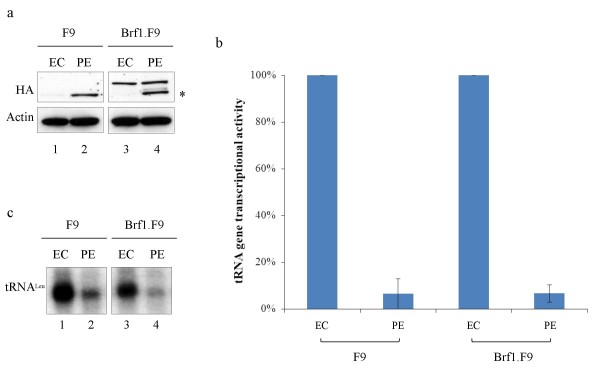
**Stable expression of Brf1 in F9 cells does not prevent down-regulation of tRNA gene transcription following differentiation**. a Western blot of whole cell extract (50 μg) from untransfected F9 cells (lanes 1 and 2) and Brf1.F9 cells (lanes 3 and 4) before (EC) or after (PE) differentiation. Blots were probed with antibodies F-7 against HA and C-11 against actin, as indicated. b Quantitative comparison of tRNA^Leu ^gene transcription in vitro using extracts (20 μg) of Brf1.F9 and untransfected F9 cells before (EC) and after (PE) differentiation. Graph shows mean from three independent experiments +/- standard deviation. c Representative example of one of the transcription assays quantified in Fig. 3b.

Although apparently not limiting, the exogenous Brf1 in our cell lines allowed us to determine if down-regulation of pol III transcription during F9 cell differentiation is dependent on the decrease in Brf1 expression that normally accompanies this transition [2]. To facilitate detection, a HA tag was included at the N-terminus of the exogenous Brf1 in Brf1.F9 cells. Immunoblotting with antibody against this tag detected not only HA-Brf1, but also a smaller protein of unknown identity, that appears during differentiation; this protein is not derived from the vector, as it is also detected in untransfected cells (Fig. 3a, lanes 1 and 2). Nevertheless, it is clear that HA-Brf1 levels are maintained in the Brf1.F9 cells after differentiation into PE (Fig. 3a, lanes 3 and 4). Despite this, tRNA gene transcriptional activity was decreased by ~10-fold in extracts of fully differentiated Brf1.F9 cells that continued to express the exogenous HA-Brf1 (Figs. 3b and 3c). This change is not significantly different from the decrease seen in untransfected F9 cells (P = 0.86). These data suggest that additional changes to the pol III machinery also occur when F9 cells differentiate.

### F9 cell differentiation is accompanied by specific changes in expression of all three TFIIIB subunits

**Figure 4:**
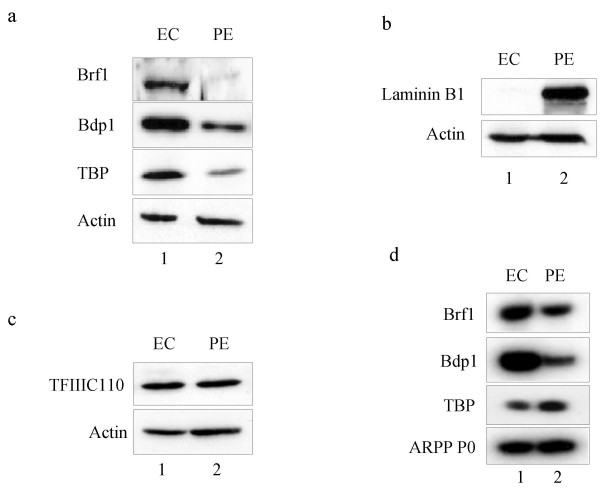
**Differentiation of F9 EC cells into PE is accompanied by specific decreases in expression of Brf1, Bdp1 and TBP proteins, as well as Brf1 and Bdp1 mRNAs**. a Western blot of whole cell extract (50 μg) from untransfected F9 cells before (EC) or after (PE) differentiation and probed with antibodies against Brf1, Bdp1, TBP and actin, as indicated. b Western blot of whole cell extract (20 μg) from untransfected F9 cells before (EC) or after (PE) differentiation and probed with antibodies against laminin B1 and actin, as indicated. c Western blot of whole cell extract (50 μg) from untransfected F9 cells before (EC) or after (PE) differentiation and probed with antibodies against TFIIIC110 and actin, as indicated. d RT-PCR analysis to compare expression of mRNAs encoding Brf1, Bdp1, TBP and ARPP P0 in undifferentiated (EC) and differentiated (PE) F9 cells, as indicated.

Western blot analysis demonstrated that not only Brf1, but also TBP and Bdp1 protein levels are markedly lower in PE cell extracts relative to undifferentiated EC cell extracts (Fig. 4a). In contrast to these decreases in the three TFIIIB subunits, the PE samples show a large increase in expression of laminin B1, an established marker of differentiation (Fig. 4b). These changes are specific, since levels of actin and TFIIIC110 (Fig. 4c) remain relatively constant when the EC and PE cell extracts are compared.

The fact that HA-Brf1 protein is expressed at unchanged levels in PE cells indicates that the observed decrease in endogenous Brf1 is unlikely to be caused by differential translation or stability. Instead, it suggests that transcription of the endogenous Brf1 gene may be regulated, a mechanism evaded by the CMV promoter that we used to express HA-Brf1. This hypothesis was supported by RT-PCR analysis, which showed that levels of endogenous Brf1 mRNA are lower following differentiation (Fig. 4d). Quantification of four experiments revealed that Brf1 is expressed in PE cells at ~62% of the level observed in undifferentiated EC cells, after normalization to ARPP P0 mRNA. Similarly, the level of Bdp1 mRNA in PE cells is ~46% of that in EC cells, on average. In contrast, TBP mRNA levels are ~72% higher in PE cells relative to EC cells. We conclude that Brf1 and Bdp1 expression is regulated at the mRNA level in differentiating F9 cells. The decrease in Brf1 mRNA expression seen by RT-PCR is less than the decrease seen in Brf1 protein by western immunoblotting. This apparent discrepancy might be explained if Brf1 protein levels respond in a non-linear fashion to the modest decrease in mRNA. In contrast to Brf1 and Bdp1, levels of TBP protein are dictated through post-transcriptional control, as shown by Perletti et al., who demonstrated the selective proteolysis of TBP when F9 cells differentiate [3].

### Both activators and repressors of TFIIIB are regulated during F9 cell differentiation

The activity of TFIIIB in mammals is subject to regulation by a variety of accessory proteins with which it is stably associated [10-12]. These include p53 and the RB family, which inhibit TFIIIB, as well as c-Myc and Erk, which activate TFIIIB. Since each of these regulators can have a profound influence on TFIIIB function, we investigated whether they are also affected by F9 cell differentiation.

**Figure 5:**
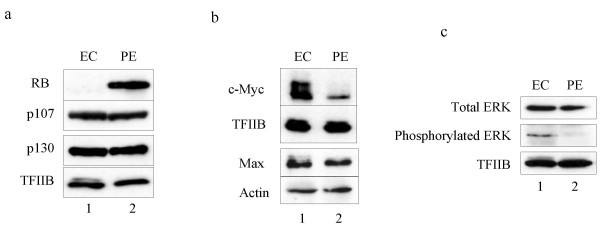
**Differentiation of F9 EC cells into PE is accompanied by specific changes in regulators of TFIIIB activity**. a Western blot of whole cell extract (20 μg) of undifferentiated (EC) and differentiated (PE) F9 cells using antibodies against RB, p107, p130 and TFIIB, as indicated. b Western blot of whole cell extract (20 μg) of undifferentiated (EC) and differentiated (PE) F9 cells using antibodies against c-Myc, Max, TFIIB and actin, as indicated. c Western blot of whole cell extract (20 μg) of undifferentiated (EC) and differentiated (PE) F9 cells using antibodies against total Erk and active (phosphorylated) Erk, as well as TFIIB, as indicated.

Several groups have shown independently that RB can bind and repress TFIIIB [13-17]. The interaction is regulated by cyclin-dependent kinases [16,18]. It results in transcriptional repression, because RB compromises the ability of TFIIIB to bind to pol III and TFIIIC [19]. Western blotting revealed a substantial increase in RB expression in PE cells (Fig. 5a). TFIIIB is also bound and inhibited by the RB-related pocket proteins p107 and p130 [20], but the levels of these change little between EC and PE cells (Fig. 5a). We also detected minimal change in p53 abundance during F9 cell differentiation (data not shown). Changes in the post-translational modification state of p53 remain a possibility, but we have not investigated these, as it is currently unknown how they may influence the ability of p53 to regulate TFIIIB.

Pol III transcription can be stimulated by the proto-oncogene product c-Myc [9,10,21,22]. Recruitment of TFIIIB to target genes is facilitated by c-Myc, an effect that involves the cofactor GCN5 and localised acetylation of histone H3 [22]. We found that expression of c-Myc decreases dramatically when F9 cells differentiate (Fig. 5B), as previously reported [23,24]. In contrast, little or no change was detected in expression of Max, the dimerisation partner of c-Myc. A second potent activator of TFIIIB is the MAP kinase Erk, which binds and phosphorylates Brf1 [25]. Although the total concentration of Erk protein is relatively constant, its active phosphorylated form is much less prevalent in PE cells (Fig. 5C). Thus, the TFIIIB remaining in these differentiated cells is likely to be deprived of two key regulators that are required for its full activity.

## Discussion

Experiments with crude phosphocellulose fractions provided the first indication that pol III transcription is down-regulated during F9 cell differentiation through inactivation of TFIIIB [1]. At the time, the composition of TFIIIB had not been determined and so molecular reagents were unavailable. The possibility therefore remained that the biochemical activity assays were in fact responding to some alternative factor, present in the same crude fractions. Once the subunits of TFIIIB were identified, regulatory studies could be conducted with a great deal more certainty. The use of specific antibodies allowed demonstration that differentiation of F9 cells is accompanied by a marked decrease in the levels of TBP and Brf1 [2]. This down-regulation of TBP has also been reported independently [3]. Nevertheless, another study suggested that although TFIIIB may become limiting and contribute to the down-regulation of transcription, it cannot account for it [5]. The authors proposed that the key regulatory event that dictates pol III output during differentiation is loss of an activity that they referred to as TFIIIC1 [5]. However, their subsequent analysis revealed that Bdp1 was present in the TFIIIC1 fractions and required for their activity [6]. Indeed, recombinant Bdp1 could substitute for the TFIIIC1 fractions in reconstituting transcription [6]. Western blotting showed a decrease in Bdp1 expression in differentiated F9 cells [6], a result that we have confirmed here with an alternative antibody and by RT-PCR. Both groups' data therefore support the original model that down-regulation of TFIIIB is responsible for the substantial decrease in pol III transcription that occurs when F9 cells differentiate. Given that the model was based on the use of extremely crude fractions, it is perhaps surprising that it has turned out to be correct.

For most pol III-transcribed genes, including those encoding tRNA and 5S rRNA, the essential components of TFIIIB are TBP, Bdp1 and Brf1 [26]. However, a subset of pol III-dependent genes use a form of TFIIIB in which Brf1 is replaced by its homologue Brf2; these all have upstream (type 3) promoters and include the genes encoding U6, 7SK and Y RNAs. We have not investigated Brf2 in this study.

Specific changes in expression of TBP- associated factors during differentiation are a feature of other transcription systems besides pol III. For example, levels of TAF_I_48 and TAF_I_95 decrease when F9 cells differentiate, causing a fall in pol I transcription [27]. The same pair of pol I-specific TAFs are down-regulated during differentiation of B cells into plasma cells [28]. These TAFs are part of the SL1 complex, which is also inactivated when U937 promyelocytic cells differentiate [29]. However, the loss of SL1 activity in U937 cells is not due to changes in the abundance of its constituent TAFs or TBP [29]. The molecular basis of this has not been established, but might reflect loss of c-Myc, which has been shown to bind and activate SL1 [30]. Down-regulated expression of some pol II-specific TAFs has also been observed during differentiation of F9 cells and C2C12 myoblasts [3,31]. Changes in TAF levels seem therefore to be used in various systems to re-programme gene transcription as cells differentiate.

Expression of the Bdp1 gene is very sensitive to TBP levels, whereas the Brf1 gene is less responsive [32]. The decreased Bdp1 mRNA in F9 PE cells may therefore be a consequence of TBP down-regulation. In contrast, an Erk inhibitor suppresses expression of Brf1, but not Bdp1 [33]. Furthermore, Brf1 expression is stimulated by c-Myc [34]. It is therefore possible that the decrease in Brf1 levels following F9 cell differentiation is caused by the observed regulation of c-Myc and/or Erk. As TBP is down-regulated by selective proteolysis in differentiating F9 cells [3], we wondered if the same applies to Brf1. However, our data argue strongly against this, since Brf1 protein levels are maintained when the gene is expressed from a constitutive promoter. Regulated proteolytic turnover has not been excluded for Bdp1, but the decrease detected in its mRNA may be sufficient to account for the down-regulation of this TAF.

The fact that forced expression of Brf1 does not restore transcription in PE cells does not mean that Brf1 control is irrelevant in this system. We interpret it as being a reflection of regulatory redundancy, where more than one control mechanism can be sufficient to achieve a phenotypic end point. Indeed, we do not believe that pol III transcription could be restored by reversing any one of the changes that we have documented. Although we have only tested this directly in the case of Brf1, we do not feel it is worth attempting further, as the prospects of success seem remote. Restoring TBP or Bdp1 levels in PE cells is unlikely to have much impact if Brf1 levels become limiting. Co-expressing all three subunits would be difficult to achieve and still would be unlikely to be effective, given the high levels of RB, which is known to bind and repress TFIIIB [13-17]. Depletion of c-Myc is sufficient to inhibit pol III transcription [10,21,30,33]. If c-Myc were introduced into PE cells, its activity would be counteracted by Max-binding repressor proteins, which are induced during differentiation [35]. Furthermore, Erk inhibition is sufficient to suppress pol III transcription [25,33] and would not be reversed by overexpressing this kinase, as its inactivation in PE cells reflects its dephosphorylation, rather than loss of Erk itself. These considerations have convinced us that attempts to restore TFIIIB activity in PE cells are unlikely to be successful.

## Conclusion

**Figure 6:**
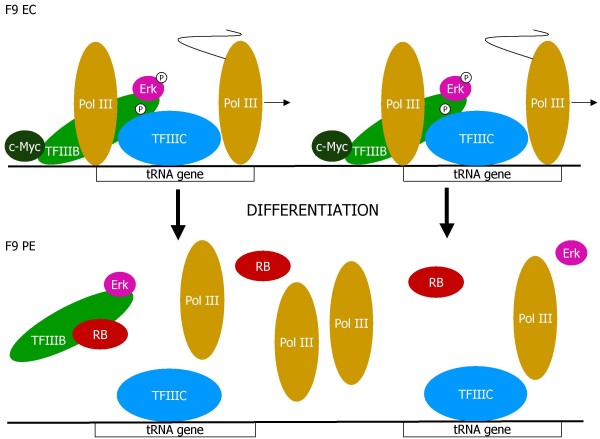
**Model of changes to the pol III transcription machinery that accompany differentiation of F9 EC cells into PE**. In EC cells, tRNA genes are transcribed very actively, reflecting the availability of TFIIIB, c-Myc and activated Erk. Differentiation is accompanied by decreased levels of TFIIIB and c-Myc, as well as inactivation of Erk and induction of RB. PE cells contain relatively little TFIIIB, which is deprived of activators and liable to inhibition by RB; as a consequence, transcription of tRNA genes is severely restricted.

In summary, differentiation of F9 EC cells is accompanied by several specific and drastic changes to TFIIIB and its associated regulators (Fig. 6). The abundance of TBP, Brf1 and Bdp1 decreases markedly. This reflects a fall in the expression of mRNAs encoding Brf1 and Bdp1, whereas TBP is regulated post-transcriptionally. The TFIIIB remaining in PE cells is deprived of c-Myc and active Erk, both of which are important for its optimal function. In addition, it must contend with highly-elevated levels of its inhibitor RB. These combined changes are more than sufficient to explain the low rates of pol III transcription that are a feature of this differentiated cell type.

## Methods

### Cell culture and stable transfection

F9 EC cells were cultured in Dulbecco's modified eagle medium containing 10% foetal bovine serum, 1 mM L-glutamine, 100 U/ml penicillin and 100 μg/ml streptomycin (all from Sigma). Full differentiation was achieved after 6-7 days by supplementing the medium with 0.1 μM retinoic acid, 1 mM dibutyryl cyclic AMP and 0.1 mM 3-isobutyl-1-methylxanthine (all from Sigma). G418 sulphate (PAA laboratories) was included at a concentration of 500 μg/ml in the medium for stably transfected cells.

Brf1.F9 and Vec.F9 lines were made by transfecting F9 EC cells with pcDNA3.1-HA-HsBrf1 [19] or pcDNA3.1-HA vector (Invitrogen), respectively, using Superfect (Qiagen). After 24 hrs, medium was supplemented with G418 sulphate (500 μg/ml). G418-resistant clones were isolated after four weeks and screened for expression of HA-Brf1 by western blotting with anti-HA antibody F-7 (Santa Cruz Biotechnology Inc).

### RNA and protein expression assays

Transcript expression was assayed by RT-PCR, using published procedures for RNA extraction and reverse transcription [36]. Primers and PCR parameters have been described for Brf1 mRNA [37], Bdp1 and TBP mRNA [38], ARPP P0 mRNA [39], tRNA^Leu ^[40] and tRNA_i_^Met ^[7]. In vitro transcription assays were conducted with whole cell extracts as previously described [41].

Protein expression was assayed by western blotting of whole cell extracts, as previously described [41]. Antibodies used were 128 against Brf1 [42], 2663 against Bdp1 [8], 4286 against TFIIIC110 [39], 9102 against Erk and 9106 against activated Erk (both from Cell Signalling Technology), and the following antibodies from Santa Cruz Biotechnology Inc: 58C9 against TBP, IF8 against RB, C-18 against p107, C-20 against p130, N-262 against c-Myc, C-17 against Max, C-18 against TFIIB, A-1 against laminin B1, C-11 against actin and F-7 against the HA tag. Quantification was performed using the TotalLab software from Nonlinear Dynamics, Newcastle, UK and normalized against actin.

## Abbreviations

(ARPP): acidic ribosomal phosphoprotein; (Bdp): B double prime; (Brf): TFIIB-related factor; (EC): embryonal carcinoma; (PE): parietal endoderm; (pol): RNA polymerase; (TAF): TBP-associated factor; (TBP): TATA-binding protein.

## Authors' contributions

All experiments were carried out by DA and LM. RJW was responsible for project management and manuscript preparation. All authors read and approved the final manuscript.
